# Electrosynthesis of Copolymers Based on 1,3,5-Tris(*N*-Carbazolyl)Benzene and 2,2′-Bithiophene and Their Applications in Electrochromic Devices

**DOI:** 10.3390/polym9100518

**Published:** 2017-10-17

**Authors:** Chung-Wen Kuo, Po-Ying Lee

**Affiliations:** Department of Chemical and Materials Engineering, National Kaohsiung University of Applied Sciences, Kaohsiung 80778, Taiwan; k40017105@gcloud.csu.edu.tw

**Keywords:** conjugated polymer, polycarbazole, electrochromic device, electrosynthesis, optical contrast, coloration efficiency

## Abstract

Poly(1,3,5-tris(*N*-carbazolyl)benzene) (PtnCz) and three copolymers based on 1,3,5-tris(*N*-carbazolyl)benzene (tnCz) and 2,2′-bithiophene (bTp) were electrochemically synthesized. The anodic P(tnCz1-bTp2) film with a tnCz/bTp feed molar ratio of 1/2 showed four colors (light orange at 0.0 V, yellowish-orange at 0.7 V, yellowish-green at 0.8 V, and blue at 1.1 V) from the neutral state to oxidized states. The optical contrast (∆*T*%) and coloration efficiency (η) of the P(tnCz1-bTp2) film were measured as 48% and 112 cm^2^∙C^−1^, respectively, at 696 nm. Electrochromic devices (ECDs) based on PtnCz, P(tnCz1-bTp1), P(tnCz1-bTp2), P(tnCz1-bTp4), and PbTp films as anodic polymer layers and poly(3,4-dihydro-3,3-dimethyl-2H-thieno[3,4-b-1,4]dioxepin) (PProDOT-Me_2_) as cathodic polymer layers were assembled. P(tnCz1-bTp2)/PProDOT-Me_2_ ECD showed three various colors (saffron yellow, yellowish-blue, and dark blue) at potentials ranging from −0.3 to 1.5 V. In addition, P(tnCz1-bTp2)/PProDOT-Me_2_ ECD showed a high ∆*T*% value (40% at 630 nm) and a high coloration efficiency (519 cm^2^∙C^−1^ at 630 nm).

## 1. Introduction

Organic redox-active materials have attracted great interest during the past decades for their potential applications in commercial electronic devices [1,2,3]. π-Conjugated polymers (CPs) have been recognized as an important class of organic redox-active materials. Recently, scientists have paid attention to the applications of CPs as electrode materials in electrochromic devices (ECDs) because CPs are capable of changing their optical properties at various voltages [4]. CPs have been extensively used for several regions, such as solar cells [5,6], catalysts [7,8,9], polymer light-emitting diodes [10,11,12], supercapacitors [13,14], sensors [15,16], and ECDs [17,18].

The most widely used CPs for electrochromic applications are polyanilines (PANIs) [19], polythiophenes (PThs) [20], polypyrroles (PPys) [21], poly(3,4-ethylenedioxythiophene)s (PEDOTs) [22,23,24], polycarbazoles (PCzs) [25], and polyindoles [26]. Thin films of these CPs were prepared using chemical synthesis or electrodeposition. PCzs have been widely investigated because of their attractive and practical opto-electrochemical properties. Carbazole units can be substituted or attached at the 3,6-, 2,7- and *N*-positions; a broad variety of aryl groups can be incorporated at the 3,6-, 2,7- and *N*-positions of carbazole units, and the opto-electronic properties of PCzs can be effectively tuned by incorporating the substituted group at various positions of PCzs [27,28,29]. PThs are one of the most precious types of CPs that may be easily modified to offer a variety of useful physicochemical and electrochemical properties [30]. PThs without other substituents are insoluble in any solvent; PEDOTs and poly(3,4-dihydro-3,3-dimethyl-2H-thieno[3,4-b-1,4]dioxepin) (PProDOT-Me_2_) contain two electron-donating oxygen atoms at the 3,4-positions of Th unit, which shows a higher solubility than that of PTh. Moreover, the band gaps of PEDOTs and PProDOT-Me_2_ are lower than that of PTh, and PEDOTs as an EC material have showed promising properties in electrochromic iris devices [22]. On the other hand, the copolymerization of specific monomers can bring about more interesting electrochemical and electrochromic properties than that of homopolymers. Accordingly, three copolymers based on 1,3,5-tris(*N*-carbazolyl)benzene (tnCz) and 2,2′-bithiophene (bTp) and two homopolymers (poly(1,3,5-tris(*N*-carbazolyl)benzene) and poly(2,2′-bithiophene)) were synthesized electrochemically in this study. 1,3,5-Tris(*N*-carbazolyl)benzene contains three carbazole units linked by a phenyl unit, which offers several polymerization locations at the 3,6-positions of three carbazole units. The electrochromic and spectroelectrochemical properties of two homopolymer films and three copolymer films were comprehensively studied. Moreover, five ECDs were fabricated using PtnCz, P(tnCz1-bTp1), P(tnCz1-bTp2), P(tnCz1-bTp4), and PbTp films as the anodic layers, and PProDOT-Me_2_ as the cathodic layer. The spectroelectrochemical characterization, coloration efficiency, switching time, and multiple cycling stability of PtnCz/PProDOT-Me_2_, P(tnCz1-bTp1)/PProDOT-Me_2_, P(tnCz1-bTp2)/PProDOT-Me_2_, P(tnCz1-bTp4)/PProDOT-Me_2_, and PbTp/PProDOT-Me_2_ ECDs were also studied.

## 2. Materials and Methods

### 2.1. Materials

ProDOT-Me_2_ was synthesized on the basis of previously published procedures [31]; bTp, tris(4-iodophenyl)amine, and LiClO_4_ were purchased from Sigma-Aldrich (St. Louis, MO, USA). Poly(methyl methacrylate) (PMMA) (*M*_w_ = 350,000), 18-crown-6, and carbazole were purchased from Acros Organics (Geel, Belgium). Acetonitrile (ACN) was purchased from Alfa Aesar (Haverhill, MA, USA) and was used as received.

### 2.2. Synthesis of 1,3,5-Tris(N-Carbazolyl)Benzene (tnCz)

A mixture of 1,3,5-tribromobenzene (69.26 mg, 0.22 mmol), carbazole (140.45 mg, 0.84 mmol), potassium carbonate (331.7 mg, 2.4 mmol), 18-crown-6 (17.44 mg, 0.066 mmol), copper bronze (139.17 mg, 2.19 mmol) and 70 mL of 1,2-dichlorobenzene was stirred under nitrogen for 36 h at 185 °C. Afterwards, 1,2-dichlorobenzene was evaporated and the remaining mixture was purified by column chromatography (silica gel; 1:2 dichloromethane (DCM)/hexane) to give 1,3,5-tris(*N*-carbazolyl)benzene with a yield of 46%. ^1^H NMR (500 MHz, dimethyl sulfoxide (DMSO)-d_6_): δ of 8.28 (d, 6H, Cz-H), 8.05 (s, 3H, phenyl-H), 7.77 (d, 6H, Cz-H), 7.52 (dd, 6H, Cz-H), and 7.34 (dd, 6H, Cz-H). Elem. anal. calcd. for C_42_H_27_N_3_: C, 87.93%; H, 4.74%; N, 7.32%. Found: C, 87.78%; H, 4.69%; N, 7.21%.

### 2.3. Electrochemical Polymerization

Electrochemical polymerizations of PtnCz, P(tnCz1-bTp1), P(tnCz1-bTp2), P(tnCz1-bTp4), and PbTp films were carried out in a three-electrode system. A platinum wire and a Ag/AgCl electrode were used as counter and reference electrodes, respectively. PtnCz, P(tnCz1-bTp1), P(tnCz1-bTp2), P(tnCz1-bTp4), and PbTp films were prepared potentiodynamically at the potential range of 0.0–1.9 V (vs. Ag/AgCl) at 100 mV∙s^−1^ for three cycles. The feed species of anodic polymer films are listed in Table 1. The active areas of PtnCz, P(tnCz1-bTp1), P(tnCz1-bTp2), P(tnCz1-bTp4), and PbTp films were 1.0 × 1.5 cm^2^.

### 2.4. Construction of Dual-Type ECDs

The electrochromic electrolyte was prepared using a mixture solution of PMMA, LiClO_4_, ACN, and DCM according to our previous procedures [32]. Five anodic layers (PtnCz, P(tnCz1-bTp1), P(tnCz1-bTp2), P(tnCz1-bTp4), and PbTp films) and a PProDOT-Me_2_ layer faced each other to assemble PtnCz/PProDOT-Me_2_, P(tnCz1-bTp1)/PProDOT-Me_2_, P(tnCz1-bTp2)/PProDOT-Me_2_, P(tnCz1-bTp4)/PProDOT-Me_2_, and PbTp/PProDOT-Me_2_ ECDs, which were separated by an electrochromic electrolyte. The length and width of the electrochromic devices were 1.5 and 1.0 cm, respectively.

### 2.5. Spectroelectrochemical and Electrochemical Characterization

The electrochemical experiments were characterized using a CHI627D electrochemical analyzer (CH Instruments, Austin, TX, USA). A platinum wire, an Indium Tin Oxide (ITO) glass (1.5 cm^2^), and an Ag/AgCl electrode were employed as the counter electrode, working electrode, and reference electrode, respectively. The spectroelectrochemical measurements were carried out using an Agilent Cary 60 UV–Visible spectrophotometer (Varian Inc., Walnut Creek, CA, USA) to monitor the UV–Visible spectra. Double potential chronoamperometry was performed with the three-electrode cell using an Agilent Cary 60 UV–Visible spectrophotometer and a CHI627D electrochemical analyzer.

## 3. Results and Discussion

### 3.1. Electrochemical Polymerizations

**Figure 1:**
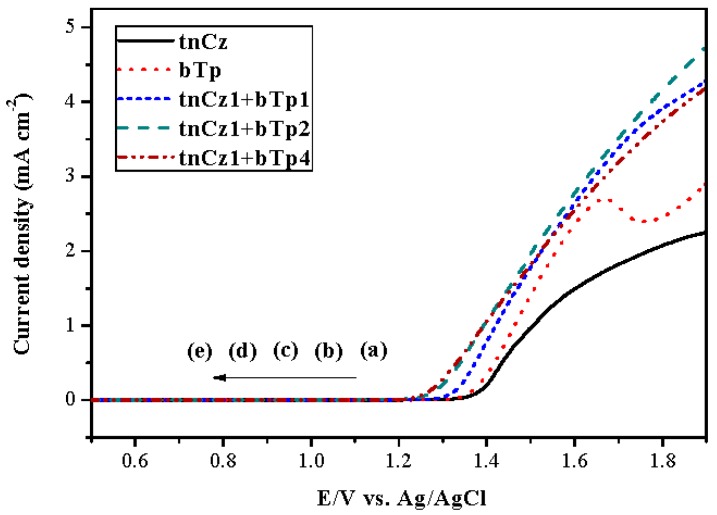
Anodic polarization curves of (**a**) 2 mM tnCz, (**b**) 4 mM bTp, (**c**) 2 mM tnCz + 2 mM bTp, (**d**) 2 mM tnCz + 4 mM bTp, and (**e**) 1 mM tnCz + 4 mM bTp in ACN/DCM (1:2, by volume) containing 0.2 M LiClO_4_ at a scan rate of 100 mV∙s^−1^.

Figure 1 shows the anodic polarization curves of the neat tnCz, the neat bTp, and the mixtures (tnCz + bTp) in ACN/DCM (1:2, by volume) solution containing 0.2 M LiClO_4_ at a constant scan rate of 100 mV∙s^−1^. The onset voltage of neat tnCz, neat bTp, tnCz1 + bTp1 (tnCz/bTp = 1:1, by feed molar ratio), tnCz1 + bTp2 (tnCz/bTp = 1:2, by feed molar ratio), and tnCz1+bTp4 (tnCz:bTp = 1:4, by feed molar ratio) were 1.36, 1.35, 1.32, 1.26, and 1.25 V, respectively. 

The *E*_onset_ of PtnCz was comparable to that of PbTp, implying that PtnCz shows a similar electron-donating ability to that of PbTp. However, P(tnCz1-bTp1), P(tnCz1-bTp2) and P(tnCz1-bTp4) showed lower *E*_onset_ values than those of PtnCz and PbTp, indicating that the *E*_onset_ of copolymers is lower than those of homopolymers. The onset potential of neat tnCz was close to that of neat bTp, demonstrating that the copolymerizations of tnCz and bTp are practicable.

**Figure 2:**
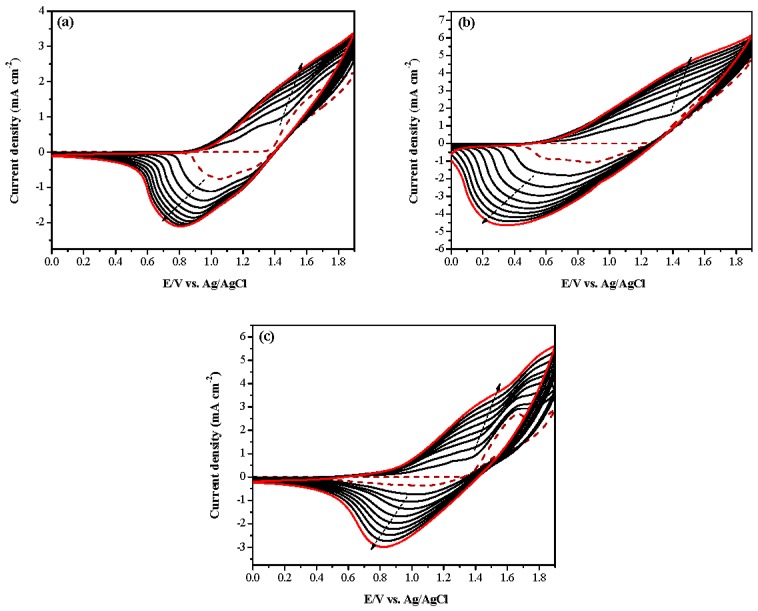
Electrochemical polymerizations of (**a**) PtnCz, (**b**) P(tnCz1-bTp2), and (**c**) PbTp in ACN/DCM (1:2, by volume) solution at 100 mV∙s^−1^ on ITO working electrode.

Figure 2a–c shows the electrosynthesis of neat tnCz, mixture (tnCz + bTp), and neat bTp in 0.2 M LiClO_4_/(ACN/DCM (1:2, by volume)) solution, respectively. The current density of cyclic voltammetry (CV) curves in Figure 2a–c increases with an increasing number of scanning cycles, implying the growth of PtnCz, P(tnCz1-bTp2), and PbTp on the ITO electrode [21]. As displayed in Figure 2a, the oxidation and reduction peaks of PtnCz, located at 1.31 and 0.98 V, respectively, are smaller than those of PbTp (the oxidation and reduction peaks of PbTp are situated at 1.68 and 1.04 V, respectively). The redox peaks of the P(tnCz1-bTp2) film shifted to lower potentials than those of PtnCz and PbTp, indicating that the copolymer gives rise to lower redox peaks than those of homopolymers. Moreover, the locations of the oxidation and reduction peaks and the waveshapes of the CV curves of the P(tnCz1-bTp2) film are different to those of the PtnCz and PbTp films, demonstrating the formation of the P(tnCz1-bTp2) film.

**Figure 3:**
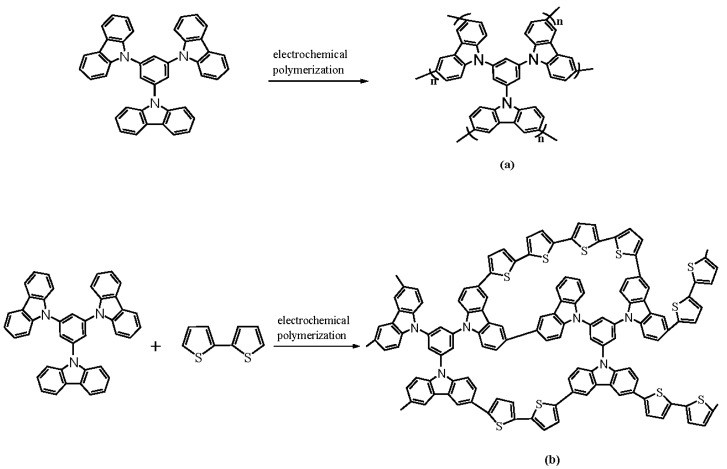
The electrochemical polymerization routes of (**a**) PtnCz and (**b**) P(tnCz1-bTp2).

**Figure 4:**
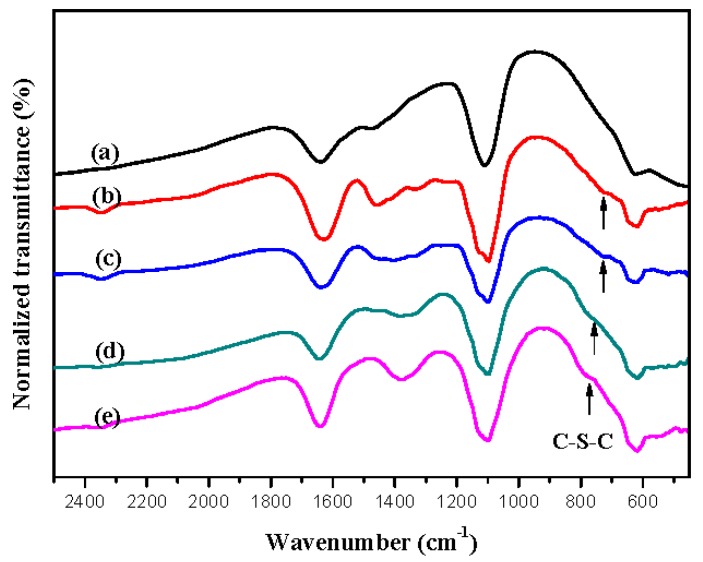
FT-IR spectra of (**a**) PtnCz, (**b**) P(tnCz1-bTp1), (**c**) P(tnCz1-bTp2), (**d**) P(tnCz1-bTp4), and (**e**) PbTp.

The electrochemical polymerization routes of PtnCz and P(tnCz1-bTp2) are shown in Figure 3. Moreover, the homopolymers (PtnCz and PbTp) and copolymers (P(tnCz1-bTp1), P(tnCz1-bTp2), and P(tnCz1-bTp4)) were further studied using Fourier transform infrared (FT-IR). As shown in Figure 4, the C–S–C characteristic peaks of the P(tnCz1-bTp1), P(tnCz1-bTp2), P(tnCz1-bTp4), and PbTp films, located at 730, 735, 760, and 783 cm^−1^, and the C–S–C characteristic peaks of P(tnCz1-bTp1), P(tnCz1-bTp2), and P(tnCz1-bTp4) are different to those of PbTp, indicating that copolymerization occurs during the electropolymerization of PtnCz and PbTp. 

**Figure 5:**
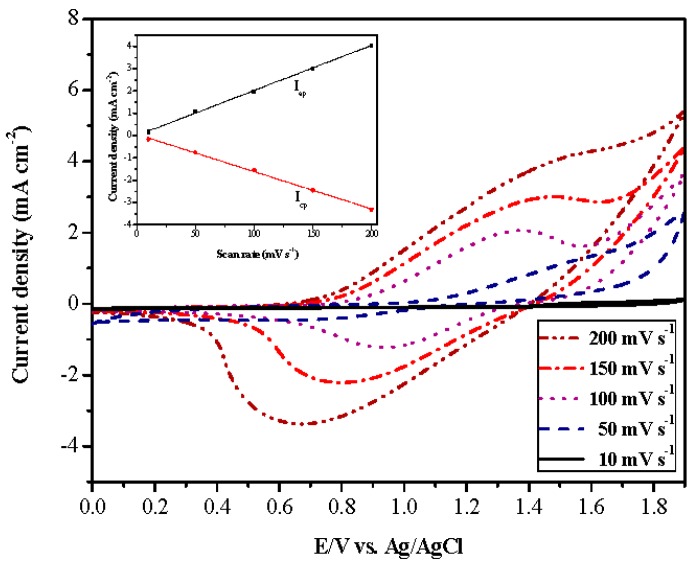
CV curves of the P(tnCz1-bTp2) film at several scan rates between 10 and 200 mV∙s^−1^ in the LiClO_4_ + ACN + DCM solution. Scan rate dependence of the P(tnCz1-bTp2) anodic and cathodic peak current densities, respectively (inset).

The as-prepared P(tnCz1-bTp2) film was also investigated at several scan rates between 10 and 200 mV∙s^−1^ in LiClO_4_/(ACN + DCM) solution. As displayed in Figure 5, the P(tnCz1-bTp2) film showed distinct redox peaks, and the current density of the reduction and oxidation peaks showed a linear relationship with the scan rate, indicating that the P(tnCz1-bTp2) film was well-adhered onto indium tin oxide conductive glass and that the reduction and oxidation processes of P(tnCz1-bTp2) film were nondiffusional processes [33].

### 3.2. Spectroelectrochemical Characterizations of PtnCz, P(tnCz-bTp), and PbTp Films

**Figure 6:**
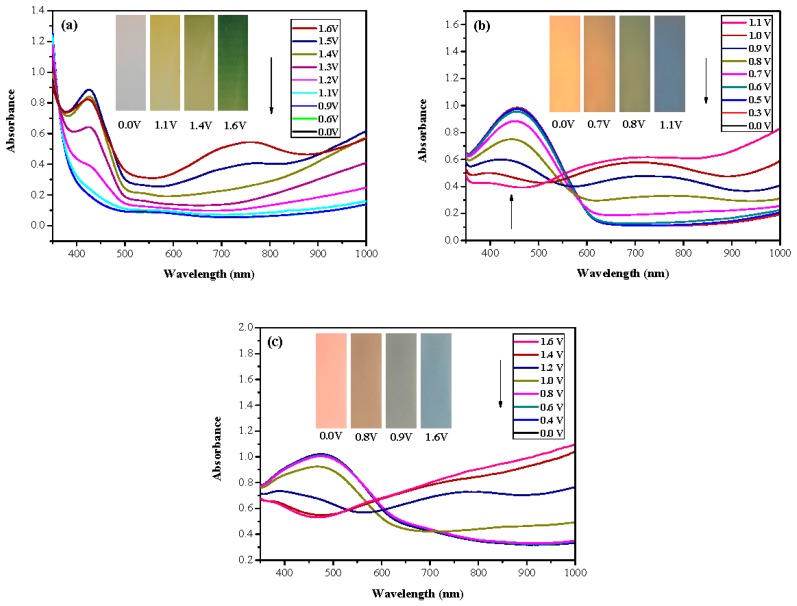
UV–Visible spectra of (**a**) PtnCz, (**b**) P(tnCz1-bTp2), and (**c**) PbTp electrodes on ITO in an ACN + DCM solution containing 0.2 M LiClO_4_.

Spectroelectrochemical characterizations of the PtnCz, P(tnCz1-bTp1), P(tnCz1-bTp2), P(tnCz1-bTp4), and PbTp films were carried out in 0.2 M LiClO_4_/(ACN + DCM) solution. Figure 6a–c shows the UV–Vis spectra of the PtnCz, P(tnCz1-bTp2), and PbTp films, respectively, at various potentials. As shown in Figure 6a, the PtnCz film does not show a distinct absorption peak in its neutral state. Nevertheless, new absorption bands appear at 430 and 750 nm after a potential of more than 1.2 V was applied, which can be ascribed to the generation of polaron and bipolaron bands for the PtnCz film [34].

The P(tnCz1-bTp2) and PbTp films show π–π* transition peaks of thiophene chains at 450 and 480 nm, respectively, in their neutral state, and the P(tnCz1-bTp2) and PbTp films show polaron and bipolaron bands at 700 and 900 nm, respectively. The PtnCz film in 0.2 M LiClO_4_/(ACN + DCM) solution was light grey in the neutral state (0.0 V), yellow in the intermediate state (1.1 V), yellowish-green in the oxidized state (1.4 V), and green in highly oxidized states (1.6 V). For the copolymer films in 0.2 M LiClO_4_/(ACN + DCM) solution, the P(tnCz1-bTp2) film was light orange in the neutral state (0.0 V), yellowish-orange (0.7 V) and yellowish-green (0.8 V) in the intermediate state, and blue in oxidized states (1.1 V). The P(tnCz1-bTp2) film exhibited different colors to PtnCz film in the oxidized states. Under similar conditions, the PbTp film in 0.2 M LiClO_4_/(ACN + DCM) solution was tangerine in the neutral state (0.0 V), yellowish-brown in the intermediate state (0.8 V), cadet blue in the oxidized state (0.9 V), and blue in highly oxidized states (1.6 V). The colorimetric parameters of the PtnCz, P(tnCz1-bTp2), and PbTp films in 0.2 M LiClO_4_/(ACN + DCM) solution are shown in Table 2.

**Figure 7:**
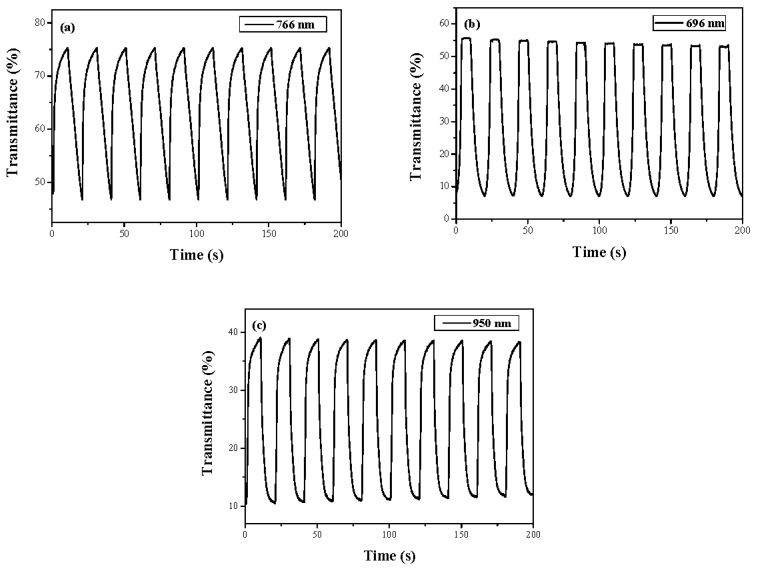
Optical transmittance change of (**a**) PtnCz, (**b**) P(tnCz1-bTp2), and (**c**) PbTp electrodes in an ACN + DCM solution containing 0.2 M LiClO_4_ with a residence time of 10 s.

The electrochromic switching of the PtnCz, P(tnCz1-bTp2), and PbTp films was examined using double-potential-step chronoamperometry [35]. Figure 7 shows the transmittance–time profiles of the PtnCz, P(tnCz1-bTp2), and PbTp films in 0.2 M LiClO_4_/(ACN + DCM) solution with a residence time of 10 s; ∆*T*, the optical density (∆OD), and the coloration efficiency (η) of the PtnCz, P(tnCz1-bTp1), P(tnCz1-bTp2), P(tnCz1-bTp4), and PbTp films in 0.2 M LiClO_4_/(ACN + DCM) solution are shown in Table 3. The ∆*T* value of the PtnCz, P(tnCz1-bTp1), P(tnCz1-bTp2), P(tnCz1-bTp4), and PbTp films were 28.6%, 45.0%, 48.0%, 36.7%, and 28.1%, respectively. The copolymers (P(tnCz1-bTp1), P(tnCz1-bTp2), and P(tnCz1-bTp4)) showed higher ∆*T* values than the homopolymers (PtnCz and PbTp) in 0.2 M LiClO_4_/(ACN + DCM) solution, implying that the copolymerization of tnCz with the bTp monomer leads to an increase in the ∆*T*_max_ value in 0.2 M LiClO_4_/(ACN + DCM) solution. The P(tnCz1-bTp2) film showed the highest ∆*T* value (48%) at 696 nm among these polymer films.

∆OD can be estimated by the following equation:

(1) $$∆\mathrm{OD}=\log\left(\frac{T_{\mathrm{ox}}}{T_{\mathrm{red}}}\right)$$

where *T*_ox_ and *T*_red_ represent the transmittance of the oxidized state and reduced state, respectively. The ∆OD value of the PtnCz, P(tnCz1-bTp1), P(tnCz1-bTp2), P(tnCz1-bTp4), and PbTp films was 0.207, 0.766, 0.895, 0.652, and 0.559, respectively, in 0.2 M LiClO_4_/(ACN + DCM) solution. Similarly to the trend of ∆*T*, the copolymers (P(tnCz1-bTp1), P(tnCz1-bTp2), and P(tnCz1-bTp4)) showed a higher ∆OD value than the homopolymers (PtnCz and PbTp) in 0.2 M LiClO_4_/(ACN + DCM) solution.

The coloration efficiency (η) can be calculated using the following equation:

(2) $$\eta =\frac{∆\mathrm{OD}}{Q_{d}}$$

where *Q*_d_ is the injected/ejected electronic charge of polymer films per active area and ∆OD is the discrepancy of optical density. As listed in Table 3, the η value of the PtnCz film at 766 nm, the P(tnCz1-bTp1) film at 680 nm, the P(tnCz1-bTp2) film at 696 nm, the P(tnCz1-bTp4) film at 689 nm, and the PbTp film at 950 nm were 103.7, 180.3, 112.0, 150.1 and 83.5 cm^2^∙C^−1^, respectively. The coloration response time (τ_c_) and bleaching response time (τ_b_) of the PtnCz, P(tnCz1-bTp1), P(tnCz1-bTp2), P(tnCz1-bTp4), and PbTp films in 0.2 M LiClO_4_/(ACN + DCM) solution are also shown in Table 3; the τ_c_ and τ_b_ values were calculated at 90% of the full-transmittance change.

### 3.3. Spectroelectrochemistry of ECDs

**Figure 8:**
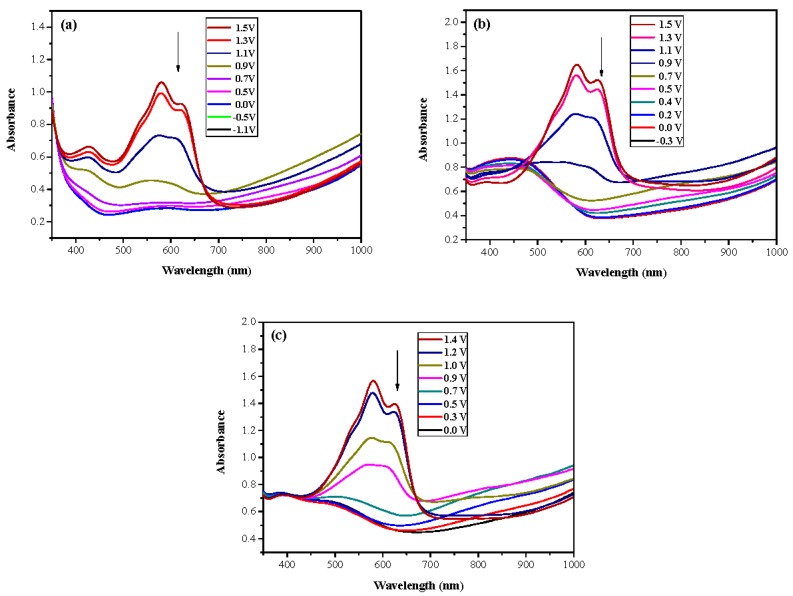
UV–Vis spectra of (**a**) PtnCz/PProDOT-Me_2_, (**b**) P(tnCz1-bTp2)/PProDOT-Me_2_, and (**c**) PbTp/PProDOT-Me_2_ electrochromic devices (ECDs).

ECDs with the configurations of PtnCz/PProDOT-Me_2_, P(tnCz1-bTp1)/PProDOT-Me_2_, P(tnCz1-bTp2)/PProDOT-Me_2_, P(tnCz1-bTp4)/PProDOT-Me_2_, and PbTp/PProDOT-Me_2_ were constructed. Figure 8a–c shows the UV-Vis spectra of PtnCz/PProDOT-Me_2_, P(tnCz1-bTp2)/PProDOT-Me_2_, and PbTp/PProDOT-Me_2_ ECDs, respectively.

At 0.0 V, the PtnCz, P(tnCz1-bTp2), and PbTp films were in the neutral state, revealing light-grey, light-orange, and tangerine colors, respectively. The PProDOT-Me_2_ film was in oxidized state, displaying a transparent blue color. Accordingly, the PtnCz/PProDOT-Me_2_, P(tnCz1-bTp2)/PProDOT-Me_2_, and PbTp/PProDOT-Me_2_ ECDs did not show an obvious absorption peak below 400 nm. Upon increasing the potential gradually, the PtnCz, P(tnCz1-bTp2), and PbTp films began to oxidize, and the PProDOT-Me_2_ film began to reduce. Therefore, new peaks at 580 and 630 nm emerged gradually, and the PtnCz/PProDOT-Me_2_, P(tnCz1-bTp2)/PProDOT-Me_2_, and PbTp/PProDOT-Me_2_ ECDs revealed a dark blue color at 1.4–1.5 V. The electrochromic photographs, colorimetric values (L*, a*, and b*), CIE chromaticity values (*x*, *y*) and CIE chromaticity diagrams of the PtnCz/PProDOT-Me_2_ and P(tnCz1-bTp2)/PProDOT-Me_2_ ECDs at various applied potentials are summarized in Table 4.

**Figure 9:**
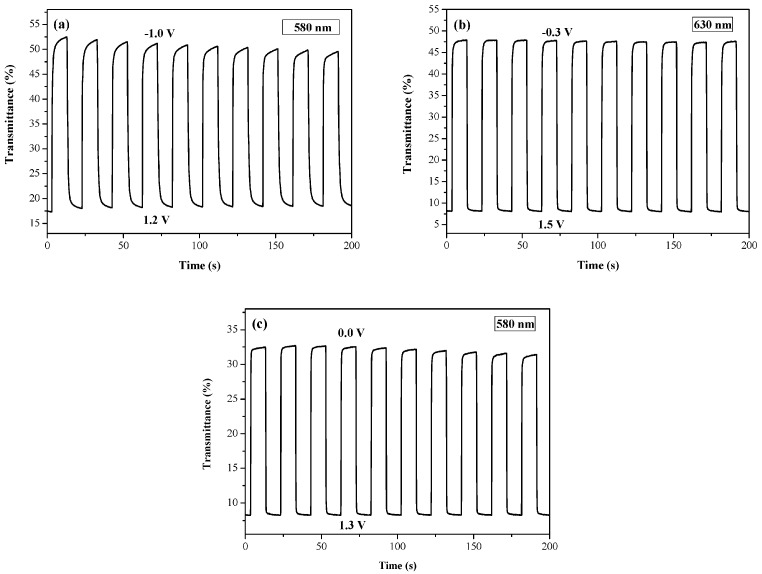
Optical transmittance change of (**a**) PtnCz/PProDOT-Me_2_, (**b**) P(tnCz1-bTp2)/PProDOT-Me_2_, and (**c**) PbTp/PProDOT-Me_2_ electrochromic devices (ECDs) with a residence time of 10 s.

Figure 9 displays the transmittance profiles as a function of time for the PtnCz/PProDOT-Me_2_, P(tnCz1-bTp2)/PProDOT-Me_2_, and PbTp/PProDOT-Me_2_ ECDs; the switching of the ECDs was monitored between −1.0 and 1.5 V with an interval of 10 s. The Δ*T*, ΔOD, η, τ_c_, and τ_b_ values of the PtnCz/PProDOT-Me_2_, P(tnCz1-bTp1)/PProDOT-Me_2_, P(tnCz1-bTp2)/PProDOT-Me_2_, P(tnCz1-bTp4)/PProDOT-Me_2_, and PbTp/PProDOT-Me_2_ ECDs estimated at the 3rd and 50th cycles are shown in Table 5. The Δ*T* value of the P(tnCz1-bTp1)/PProDOT-Me_2_, P(tnCz1-bTp2)/PProDOT-Me_2_, P(tnCz1-bTp4)/PProDOT-Me_2_ ECDs was larger than that of the PbTp/PProDOT-Me_2_ ECD, and the Δ*T* value of the P(tnCz1-bTp1)/PProDOT-Me_2_ and P(tnCz1-bTp2)/PProDOT-Me_2_ ECDs showed a larger Δ*T* value than that of the PtnCz/PProDOT-Me_2_ ECD, indicating the incorporation of copolymers as the anodic layers gave rise to a higher Δ*T* than those of the homopolymers (PtnCz and PbTp). The Δ*T* value of the PtnCz/PProDOT-Me_2_, P(tnCz1-bTp1)/PProDOT-Me_2_, P(tnCz1-bTp2)/PProDOT-Me_2_, P(tnCz1-bTp4)/PProDOT-Me_2_, and PbTp/PProDOT-Me_2_ ECDs was greater than that of the PtnCz, P(tnCz1-bTp1), P(tnCz1-bTp2), P(tnCz1-bTp4) and PbTp films, respectively, in 0.2 M LiClO_4_/(ACN + DCM) solution; this could be accredited to ECDs containing anodic and cathodic layers but there being no complementary electrode for PtnCz, P(tnCz1-bTp1), P(tnCz1-bTp2), P(tnCz1-bTp4) and PbTp films in a solution. The τ_b_ and τ_c_ values of the PtnCz/PProDOT-Me_2_, P(tnCz1-bTp1)/PProDOT-Me_2_, P(tnCz1-bTp2)/PProDOT-Me_2_, P(tnCz1-bTp4)/PProDOT-Me_2_, and PbTp/PProDOT-Me_2_ ECDs were smaller than those of the PtnCz, P(tnCz1-bTp1), P(tnCz1-bTp2), P(tnCz1-bTp4), and PbTp films, respectively, in 0.2 M LiClO_4_/(ACN + DCM) solution, implying that the ECDs changed color faster from the bleached to the colored states and from the colored to the bleached states than did the PtnCz, P(tnCz1-bTp1), P(tnCz1-bTp2), P(tnCz1-bTp4), and PbTp films in 0.2 M LiClO_4_/(ACN + DCM) solution.

The comparisons of Δ*T*_max_ and η for the reported ECDs are summarized in Table 6; the P(tnCz1-bTp2)/PProDOT-Me_2_ ECD showed a higher Δ*T*_max_ than that reported for the PdNCz/PEDOT [36], PbmCz/PEDOT [37], PHCz/PEDOT [38], P(dNCz-Hcp)/PEDOT [39], P(dNCz-bT)/PEDOT [40], and P(Cz4-6CIn1)/PProDOT-Me_2_ ECDs [41], and the Δ*T*_max_ value of the P(tnCz1-bTp2)/PProDOT-Me_2_ ECD as comparable to that of the P(BCz-ProDOTme)/PEDOT-PSS ECD [42]. In addition, the Δ*T* value of the P(tnCz1-bTp2) ECD was lower than that reported for a non-mechanical microiris based on viologen and phenazine complementary electrochromic materials [43]. On the other hand, the P(tnCz1-bTp2)/PProDOT-Me_2_ ECD showed a higher η value at 630 nm than was reported for the PHCz/PEDOT [38], P(dNCz-Hcp)/PEDOT [39], P(dNCz-bT)/PEDOT [40], P(Cz4-6CIn1)/PProDOT-Me_2_ [41], and P(BCz-ProDOTme)/PEDOT-PSS ECDs [42]. The high Δ*T*_max_ and η values of the P(tnCz1-bTp2)/PProDOT-Me_2_ ECD makes P(tnCz1-bTp2) a potential electrochromic material for ECD applications.

### 3.4. Open Circuit Memory of Electrochromic Devices

**Figure 10:**
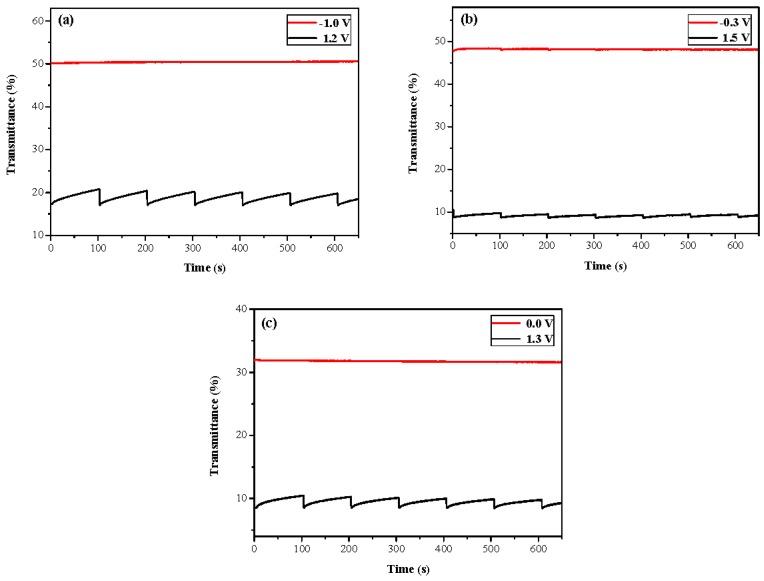
Optical memory of the (**a**) PtnCz/PProDOT-Me_2_, (**b**) P(tnCz1-bTp2)/PProDOT-Me_2_, and (**c**) PbTp/PProDOT-Me_2_ electrochromic devices (ECDs).

The ability to maintain bleached and colored states in the open circuit of the PtnCz/PProDOT-Me_2_, P(tnCz1-bTp2)/PProDOT-Me_2_, and PbTp/PProDOT-Me_2_ ECDs was monitored at a specific wavelength as a function of the time in the bleached and colored states by applying the voltage for 1 s for each 200 s time interval. As shown in Figure 10, the P(tnCz1-bTp2)/PProDOT-Me_2_ ECD showed good optical memories in the neutral state of the P(tnCz1-bTp2) film; almost no transmittance change in the neutral state was observed. In the oxidation state of the P(tnCz1-bTp2) film, the P(tnCz1-bTp2)/PProDOT-Me_2_ ECD was rather less stable than the P(tnCz1-bTp2) film in the neutral state, but the transmittance change was less than 3% in the oxidation state of the P(tnCz1-bTp2) film, implying that the P(tnCz1-bTp2)/PProDOT-Me_2_ ECD showed a reasonable open-circuit memory.

### 3.5. Switching Stability of Electrochromic Devices

**Figure 11:**
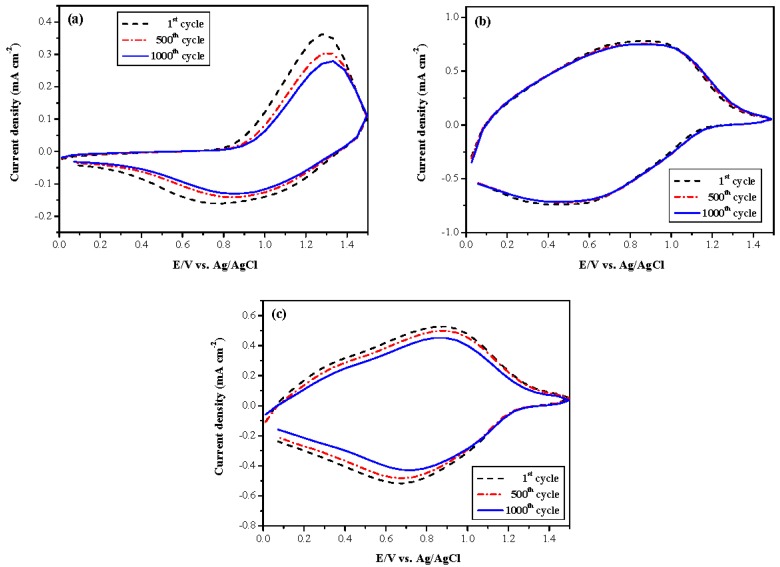
CVs of (**a**) PtnCz/PProDOT-Me_2_, (**b**) P(tnCz1-bTp2)/PProDOT-Me_2_, and (**c**) PbTp/PProDOT-Me_2_ electrochromic devices (ECDs) with a scan rate of 500 mV∙s^−1^ between 1st and 1000th cycles.

The switching stability of the PtnCz/PProDOT-Me_2_, P(tnCz1-bTp2)/PProDOT-Me_2_, and PbTp/PProDOT-Me_2_ ECDs for multiple cycles was measured using CV at voltages between 0.0 and 1.5 V. As displayed in Figure 11, 81.7%, 99.7%, and 92.4% electroactivity was retained after 500 cycles for the PtnCz/PProDOT-Me_2_, P(tnCz1-bTp2)/PProDOT-Me_2_, and PbTp/PProDOT-Me_2_ ECDs, respectively, and 73.3%, 99.3%, and 81.4% electroactivity was retained after 1000 cycles for the PtnCz/PProDOT-Me_2_, P(tnCz1-bTp2)/PProDOT-Me_2_, and PbTp/PProDOT-Me_2_ ECDs, respectively. The P(tnCz1-bTp2)/PProDOT-Me_2_ ECD employing the P(tnCz1-bTp2) copolymer as an anodic polymer layer shows better multiple switching stability than the homopolymers as anodic layers for the PtnCz/PProDOT-Me_2_ and PbTp/PProDOT-Me_2_ ECDs.

## 4. Conclusions

Two anodic homopolymer films (PtnCz and PbTp) and three anodic copolymer films (P(tnCz1-bTp1), P(tnCz1-bTp2), and P(tnCz1-bTp4)) were prepared using electrochemical polymerization. The electrochromic studies of anodic polymer films in 0.2 M LiClO_4_/(ACN + DCM) solution exhibited that the P(tnCz1-bTp2) film was light-orange in the neutral state, yellowish-orange in the intermediate state, yellowish-green in the oxidized state, and blue in highly oxidized states. Electrochromic switching characterizations of anodic polymer films in 0.2 M LiClO_4_/(ACN + DCM) solution showed high Δ*T*_max_ values for P(tnCz1-bTp2) (48% at 696 nm) and P(tnCz1-bTp1) (45% at 680 nm). The electrochromic behaviors of five ECDs (PtnCz/PProDOT-Me_2_, P(tnCz1-bTp1)/PProDOT-Me_2_, P(tnCz1-bTp2)/PProDOT-Me_2_, P(tnCz1-bTp4)/PProDOT-Me_2_, and PbTp/PProDOT-Me_2_ ECDs) were characterized. The P(tnCz1-bTp2)/PProDOT-Me_2_ ECD revealed a high Δ*T*_max_ value (40% at 630 nm), a high η value (539.4 cm^2^∙C^−^^1^ at 630 nm), a satisfactory open-circuit memory, and a satisfactory redox cycling stability. On the basis of the above results, the P(tnCz1-bTp2)/PProDOT-Me_2_ ECD is a candidate for applications in self-dimming mirrors and energy-saving windows.

## Figures and Tables

**Table 1:** Feed species of anodic layers (a)–(e).

Electrodes	Anodic Polymer	Feed Species of Anodic Polymer	Feed Molar Ratio of Anodic Polymer
(a)	PtnCz	2 mM tnCz	Neat tnCz
(b)	P(tnCz1-bTp1)	2 mM tnCz + 2 mM bTp	1:1
(c)	P(tnCz1-bTp2)	2 mM tnCz + 4 mM bTp	1:2
(d)	P(tnCz1-bTp4)	1 mM tnCz + 4 mM bTp	1:4
(e)	PbTp	4 mM bTp	Neat bTp

**Table 2:** Colorimetric values (L*, a*, and b*), CIE (Commission Internationale de I'Eclairage) chromaticity values (*x*, *y*) and diagrams of the PtnCz, P(tnCz1-bTp2), and PbTp films at various applied potentials.

Polymers	Potential (V)	L*	a*	b*	*x*	*y*	Diagrams
PtnCz	0.0	92.77	1.22	7.05	0.4567	0.4125	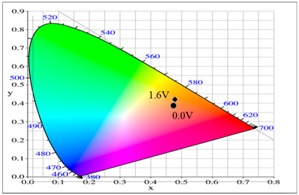
	1.1	91.69	0.96	9.62	0.4589	0.4152	
	1.2	89.86	0.48	19.25	0.4676	0.4243	
	1.3	87.25	0.00	31.83	0.4785	0.4356	
	1.4	83.10	–1.91	36.46	0.4802	0.4431	
	1.5	78.08	–5.35	33.74	0.4728	0.4480	
	1.6	73.61	–7.75	25.77	0.4616	0.4465	
P(tnCz1-bTp2)	0.0	71.78	34.58	54.01	0.5661	0.3964	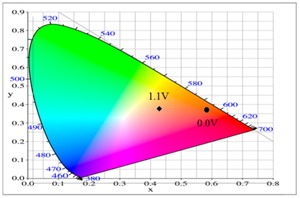
	0.6	72.11	32.68	52.59	0.5614	0.3989	
	0.7	72.03	25.51	47.02	0.5445	0.4078	
	0.8	70.79	12.38	33.90	0.5098	0.4209	
	0.9	67.83	–1.02	14.63	0.4640	0.4260	
	1.0	64.23	–7.63	–2.57	0.4274	0.4161	
	1.1	62.30	–7.98	–12.05	0.4119	0.4029	
PbTp	0.0	51.45	21.71	23.34	0.5357	0.3929	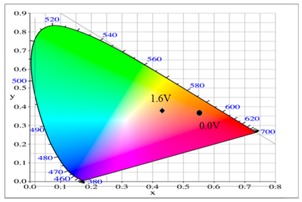
	0.6	51.01	20.62	21.90	0.5318	0.3936	
	0.8	51.01	20.34	21.58	0.5307	0.3939	
	0.9	55.79	19.12	24.19	0.5229	0.4000	
	1.2	57.79	–2.40	8.42	0.4543	0.4231	
	1.4	55.13	–7.32	–7.38	0.4177	0.4089	
	1.6	54.83	–7.08	–9.69	0.4143	0.4047	

**Table 3:** Electrochromic characterizations of electrodes.

Electrodes	λ (nm)	*T*_ox_ (%)	*T*_red_ (%)	∆*T* (%)	Contrast (%) [23]	∆OD	*Q*_d_ (mC∙cm^−2^)	η (cm^2^∙C^−1^)	τ_c_ (s)	τ_b_ (s)
PtnCz	766	46.7	75.3	28.6	23.4	0.207	2.000	103.7	5.5	4.5
P(tnCz1-bTp1)	680	9.3	54.3	45.0	70.8	0.766	4.250	180.3	5.0	2.5
P(tnCz1-bTp2)	696	7.0	55.0	48.0	77.4	0.895	7.986	112.0	6.0	4.0
P(tnCz1-bTp4)	689	10.5	47.2	36.7	63.6	0.652	4.346	150.1	5.8	3.0
PbTp	950	10.7	38.8	28.1	56.8	0.559	6.696	83.5	5.5	4.5

**Table 4:** Electrochromic photographs, colorimetric values (L*, a*, and b*), CIE chromaticity values (*x*, *y*) and diagrams of the PtnCz/PProdot-Me_2_ and P(tnCz1-bTp2)/PProdot-Me_2_ electrochromic devices (ECDs) at various applied potentials.

ECDs	Potential (V)	Photographs	L*	a*	b*	*x*	*y*	Diagrams
PtnCz/PProdot-Me2	−1.1	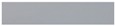	77.89	−0.64	−2.39	0.444	0.406	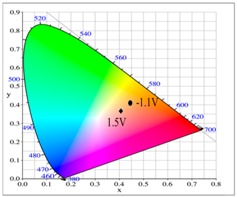
	0.0	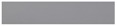	77.78	−0.67	−2.42	0.444	0.406	
	0.9	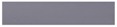	67.00	2.70	1.82	0.456	0.406	
	1.3	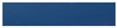	45.40	1.10	−21.68	0.407	0.366	
	1.5	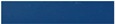	43.60	1.45	−22.35	0.405	0.362	
P(tnCz1-bTp2)/PProdot-Me2	−0.3	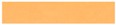	62.89	16.85	31.86	0.525	0.409	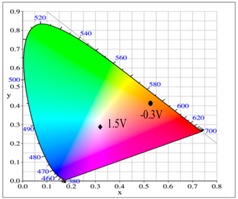
	0.4	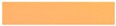	62.34	12.16	27.43	0.511	0.414	
	0.7	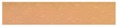	58.41	6.68	18.02	0.489	0.416	
	1.1	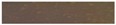	32.66	1.51	−21.96	0.396	0.351	
	1.5	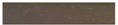	25.34	0.30	−41.33	0.318	0.284	

**Table 5:** Electrochromic characterizations of electrochromic devices (ECDs).

ECDs	*N*	*T*_ox_ (%)	*T*_red_ (%)	*∆T* (%)	Contrast (%)	∆OD	Q_d_ (mC∙cm^−2^)	η (cm^2^∙C^−1^)	τ_c_ (s)	τ_b_ (s)
PtnCz/PProdot-Me_2_ (580 nm) ^a^	3	18.2	51.4	33.2	47.7	0.450	0.859	523.9	2.5	0.5
	50	19.5	44.0	24.5	38.6	0.353	0.620	569.4	1.0	1.0
P(tnCz1-bTp1)/PProdot-Me_2_ (582 nm) ^a^	3	7.1	42.1	35.0	71.1	0.773	1.596	484.3	0.5	0.5
	50	7.5	39.0	31.5	67.7	0.716	1.252	571.8	2.0	2.0
P(tnCz1-bTp2)/PProdot-Me_2_ (630 nm) ^a^	3	8.0	48.0	40.0	71.4	0.778	1.500	518.8	0.8	0.3
	50	7.4	46.0	38.6	72.3	0.793	1.470	539.4	0.7	0.5
P(tnCz1-bTp4)/PProdot-Me_2_ (624 nm) ^a^	3	11.5	39.2	27.7	54.6	0.532	0.953	558.7	0.5	0.5
	50	9.6	32.3	22.7	54.2	0.526	0.981	537.1	0.5	0.7
PbTp/PProdot-Me_2_ (580 nm) ^a^	3	8.2	32.6	24.4	59.8	0.599	1.106	541.6	0.5	0.5
	50	8.3	28.9	20.6	55.4	0.542	0.908	596.6	0.5	0.5

^a^ The selected applied wavelength for the ECDs.

**Table 6:** Coloration efficiencies and transmission changes of electrochromic devices (ECDs).

ECD Configuration	Δ*T*_max_ (%)	η_max_ (cm^2^∙C^−1^)	Reference
PdNCz/PEDOT	19 (550 nm)	—	[36]
PbmCz/PEDOT	35 (620 nm)	—	[37]
PHCz/PEDOT	23 (623 nm)	290	[38]
P(dNCz-Hcp)/PEDOT	39.8 (628 nm)	319.98	[39]
P(dNCz-bT)/PEDOT	28.6 (700 nm)	234	[40]
P(Cz4-6CIn1)/PProDOT-Me_2_	32 (575 nm)	372.7	[41]
P(BCz-ProDOTme)/PEDOT-PSS	41 (642 nm)	417	[42]
P(tnCz1-bTp2)/PProdot-Me_2_	40 (630 nm)	539	This work
